# Everyday Financial Functioning of People with Alcohol Use Disorder

**DOI:** 10.1192/j.eurpsy.2025.957

**Published:** 2025-08-26

**Authors:** A.-M. Ariesen, L. Tucha, A. Hope, A. B. Fuermaier, O. Tucha, J. Koerts

**Affiliations:** 1 Clinical and Developmental Neuropsychology, University of Groningen, Groningen, Netherlands; 2 Psychiatry and Psychotherapy, University Medical Center Rostock, Rostock, Germany; 3 Psychology, National University of Ireland, Maynooth, Ireland

## Abstract

**Introduction:**

Alcohol Use Disorder (AUD) and its comorbidities can have a tremendous negative impact on various activities of daily living, including the capability to manage one’s finances. Adequate financial functioning is essential for an individual’s health and well-being and is key to leading an autonomous and independent life. Problems with financial functioning can have far-reaching personal and legal consequences, and may lead to financial insecurity or poverty, financial victimisation, placement under guardianship, and reduced opportunities for social and societal participation.

**Objectives:**

To evaluate the financial situation and the strengths and weaknesses in the everyday financial functioning of individuals with AUD.

**Methods:**

The financial situation and financial performance of an AUD group (n = 52) were compared to a control group (CG) (n = 95), using the Financial Performance Scale (FiPS). In addition, associations between financial performance and everyday contextual factors (i.e., income, depressive symptoms (i.e., Beck Depression Scale - II), social support (i.e., Brief Perceived Social Support Questionnaire)) were explored.

**Results:**

As compared to the CG, the AUD group reported to have a significantly poorer financial situation, including lower income levels, more frequent debts, and fewer savings. Furthermore, the AUD group reported a significantly poorer overall financial performance (FiPS total score) than the CG, and significant group differences were observed for relatively complex financial tasks, such as financial goal setting and doing tax returns. The difficulties in financial performance of the AUD group were, however, considered as relatively mild, since most aspects of financial performance (i.e., FiPS item scores) did not differ between groups. In the total sample, a better financial performance was significantly associated with a higher income, more perceived social support, and fewer depressive symptoms.

**Conclusions:**

Individuals with AUD reported a poorer financial situation and more difficulties with performing complex financial tasks compared to controls. These reported weaknesses may stem from cognitive and affective impairments associated with AUD, as well as from a scarcity of financial resources. Since a vicious cycle may exist between financial problems and AUD symptoms, it is relevant to enhance the financial well-being of those individuals with AUD who experience financial difficulties.

**Disclosure of Interest:**

None Declared

